# Ki67 Gene Expression is Associated with Immune Cell Infiltration and Neoadjuvant Chemotherapy Response in ER+/HER2− Breast Cancer

**DOI:** 10.1245/s10434-026-19620-2

**Published:** 2026-04-10

**Authors:** Kohei Chida, Rongrong Wu, Kei Kawashima, Abigail Grapes, Li Yan, Itaru Endo, Takashi Ishikawa, Kenichi Hakamada, Kazuaki Takabe

**Affiliations:** 1 Department of Surgical Oncology, Roswell Park Comprehensive Cancer Center, Buffalo, NY USA; 2 Department of Gastroenterological Surgery, Hirosaki University Graduate School of Medicine, Hirosaki, Japan; 3 Department of Breast Surgery and Oncology, Tokyo Medical University, Tokyo, Japan; 4 Department of Gastroenterological Surgery, Yokohama City University Graduate School of Medicine, Yokohama, Kanagawa Japan; 5 Department of Surgery, University at Buffalo Jacobs School of Medicine and Biomedical Sciences, The State University of New York, Buffalo, NY USA; 6 Department of Biostatistics and Bioinformatics, Roswell Park Comprehensive Cancer Center, Buffalo, NY USA; 7 Department of Immunology, Roswell Park Comprehensive Cancer Center, Buffalo, NY USA; 8 Division of Digestive and General Surgery, Niigata University Graduate School of Medical and Dental Sciences, Niigata, Japan; 9 Department of Breast Surgery, Fukushima Medical University School of Medicine, Fukushima, Japan; 10 Department of Breast Surgery, Roswell Park Comprehensive Cancer Center, Buffalo, NY USA

**Keywords:** Ki67, Breast cancer, Gene expression, Biomarker, Transcriptome, Signaling

## Abstract

**Background:**

Ki67 is one of the most widely used markers of cell proliferation. However, current measurements using immunohistochemistry (IHC) are limited by interobserver variability and lack of standardized cutoffs. To address this, we investigated the association of Ki67 gene (*MKI67*) expression with biological features and treatment response in estrogen receptor-positive (ER+) breast cancer (BC), the most common subtype.

**Patients and Methods:**

We analyzed 5036 patients with ER+/HER2− BC across 11 independent cohorts with tumor transcriptomes and clinical data. Patients with *MKI67* expression in the top 20% were defined as the high-expression group on the basis of prior IHC-based thresholds.

**Results:**

*MKI67* expression correlated with Nottingham histologic grade and proliferation score, and consistently enriched all the hallmark cell proliferation-related gene sets across TCGA, METABRIC, and SCAN-B cohorts. High *MKI67* expression trended toward worse survival but achieved statistical significance only in the METABRIC cohort. *MKI67* high ER+/HER2− BC was associated with increased mutation rates, fraction altered, homologous recombination deficiency and intratumoral genomic heterogeneity. *MKI67* expression was associated with infiltration of Th1 and Th2 cells as well as M1 and M2 macrophages across three cohorts. Conversely, *MKI67* low ER+/HER2− BC was enriched for Hypoxia, Coagulation, Epithelial-to-Mesenchymal Transition, NOTCH, Hedgehog, TGF-beta, and KRAS-signaling gene sets. *MKI67* high ER+/HER2− BC was associated with a higher pathological complete response (pCR) rate in four of the eight neoadjuvant chemotherapy cohorts.

**Conclusions:**

High *MKI67* expression identifies highly proliferative tumors that are associated with genomic instability and immune activity and is associated with higher pCR rates following neoadjuvant chemotherapy in ER+/HER2− BC.

**Supplementary Information:**

The online version contains supplementary material available at 10.1245/s10434-026-19620-2.

Sustained proliferative signaling is one of the hallmarks of cancer,^1^ and Ki67 is a nuclear protein that is most commonly used as a marker for cell proliferation.^2^^,^^3^ Ki67 levels can differentiate highly proliferative tumors from more indolent ones, particularly in estrogen receptor-positive (ER+) breast cancer (BC).^4^ Multiple studies have shown that high Ki67 is associated with greater response to neoadjuvant chemotherapy (NAC),^5^^–^^7^ but with worse long-term survival.^8^^–^^10^ Indeed, Ki67 levels greater than 20% were associated with axillary lymph node downstaging with NAC in ER+/HER2− BC,^11^ and high Ki67 index after neoadjuvant endocrine therapy was associated with worse survival in early ER+/HER2− BC.^12^

Ki67 is frequently assessed in clinical practice using immunohistochemistry (IHC). Although international working groups have proposed guidelines for scoring and reporting to standardize its use,^2^^,^^8^ significant challenges remain. Automated digital image analysis and artificial intelligence-based scoring are emerging as promising tools to reduce observer-dependent variability, but these technologies require further validation and are not yet widely implemented in clinical practice.^5^ The American Society of Clinical Oncology (ASCO) acknowledges Ki67 as a prognostic biomarker but advises against relying on it for routine treatment decisions due to variability in interpretation^5^ and the absence of universally accepted cutoffs that define positivity.^13^^–^^17^ To date, its use to guide therapy remains investigational except in select contexts such as eligibility criteria for adjuvant abemaciclib.^13^

In contrast, the transcriptomic measurement of *MKI67*, the gene encoding Ki67, has emerged as a potentially more consistent and objective method of evaluating cancer cell proliferation. Prior studies have compared *MKI67* mRNA quantification by reverse transcription-quantitative polymerase chain reaction (RT-qPCR)^18^^,^^19^ or microarray^20^ with Ki67 protein assessment by IHC, demonstrating that transcriptomic approaches may offer improved analytical reproducibility and broader dynamic range, with less observer-dependent variability. Unlike IHC-based assessments, transcriptomic data provide a quantitative readout and can be readily integrated with other genomic features to offer a more comprehensive view of tumor biology.^21^ However, prior reports on BC have been constrained by single cohorts with limited external validation.

Utilizing multiple independent large cohorts, we examined *MKI67* gene expression to assess its association with treatment response and survival outcomes in ER+/HER2− BC, the most common subtype. Additionally, we aimed to characterize the biological features associated with *MKI67* expression, such as immune cell infiltration as well as other cell types within the tumor microenvironment.

## Patients and Methods

### Data Acquisition

A total of 5036 patients with ER+/HER2− BC were included in the analysis. Three large independent cohorts: METABRIC (*n *= 1354),^22^ SCAN-B (*n *= 2277),^23^ and The Cancer Genome Atlas (TCGA, *n *= 583)^24^ were used to obtain clinical, pathological, and gene expression data. The data retrieval was carried out through cBioPortal.^25^^–^^27^ We used the normalized mRNA sequencing data converted to HUGO symbols (data_RNA_Seq_v2_expression_median.txt) and performed log2 transformation. Similarly, for the METABRIC cohort, microarray RNA expression data annotated with HUGO symbols (data_mrna_agilent_microarray.txt) were downloaded from cBioPortal and used after log2 transformation. For the SCAN-B cohort, clinical data were retrieved using the R package GEOquery (GSE96058), while the normalized data converted to HUGO symbols were downloaded directly from the NCBI Gene Expression Omnibus (GEO) database. To analyze clinical outcomes related to NAC, eight independent cohorts were included: Popovici et al. (GSE20194, *n* = 140),^28^ Hatzis et al. (GSE25066, *n* = 274),^29^ Tabchy et al. (GSE20271, *n* = 89),^30^ Ronde et al. (GSE34138, *n* = 119),^31^ Prat et al. (GSE50948, *n* = 25),^32^ Esserman et al. (GSE22226, *n* = 41),^33^ Chen et al. (GSE163882, *n* = 69),^19^ and Wolf et al. (GSE180962, *n* = 65).^34^ Across cohorts, NAC regimens consisted primarily of anthracycline- and/or taxane-based chemotherapy. Detailed chemotherapy regimen for each cohort is summarized in Supplementary Table 1. Clinical, pathological, and gene expression data for these cohorts were obtained from the GEO repository of the United States National Institutes of Health (https://www.ncbi.nlm.nih.gov/geo/) using the GEOquery package in R. For the microarray datasets (GSE20194, GSE25066, GSE20271, GSE34138, GSE50948, and GSE22226), probes were converted to HUGO symbols using the specific GPL platform for each cohort. When multiple probes mapped to the same gene, the mean expression value was calculated. For GSE180962, normalized data already assigned to HUGO symbols were downloaded directly from the GEO database, and the mean value was used for duplicate transcripts. Only the control group was utilized for the analysis of this cohort. For GSE163882, normalized RNA expression data were downloaded from GEO and annotated from Ensembl IDs to HUGO symbols using the R package *biomaRt* (accessed November 2021 on the basis of the GRCh38 reference genome). Duplicate transcripts were averaged as described for the other cohorts. All datasets were downloaded in November 2021. All data utilized in this study were obtained from publicly available databases, thus institutional review board (IRB) approval was not required.

### Gene Set Enrichment Analysis (GSEA)

Functional enrichment analysis based on *MKI67* expression levels was performed by gene set enrichment analysis (GSEA) using the Hallmark collection from the Molecular Signatures Database (MSigDB, https://www.gsea-msigdb.org)^35^ with publicly available software from the Broad Institute (http://software.broadinstitute.org/gsea/index.jsp). The analysis was conducted in a pre-ranked manner on the basis of log2 fold change of gene expression, comparing the high and low *MKI67* expression groups. Statistical significance was defined as a false discovery rate (FDR) < 0.25, and the normalized enrichment score (NES) was used to evaluate the strength of the correlation within each gene set (https://docs.gsea-msigdb.org/#GSEA/GSEA_User_Guide/#false-discovery-rate-fdr). The high *MKI67* expression group was defined as the top 20% of tumors ranked by *MKI67* expression, with the remaining 80% classified as the low expression group. This cutoff was chosen on the basis of prior IHC-based thresholds^11^ and a previous study demonstrating that the top 20% threshold yielded the strongest prognostic correlations when applied to tumor microarrays.^20^ This rank-based, within-cohort approach was intentionally used to mitigate differences in expression scaling and normalization across platforms.

### Composition of Immune Cells, Cytolytic Activity Score (CYT), and Immune-Related Scores

The xCell algorithm,^36^ a gene signature-based computational method that estimates the composition of 64 immune and stromal cell types from bulk tumor transcriptomic data, was applied to estimate immune cell infiltration in the tumor microenvironment (TME). The immune cytolytic activity (CYT) score was defined as the geometric mean of granzyme A (*GZMA*) and perforin (*PRF1*), following the method described by Rooney et al.^37^ Tumor transcriptome-based estimates of genomic instability, such as homologous recombination deficiency (HRD), tumor immunogenicity-related features such as neoantigen load, mutation rate, intratumor heterogeneity, and tumor proliferation score, were obtained from the publication by Thorsson et al.^38^

### Statistical Analysis

Kruskal–Wallis, Mann–Whitney *U*, or Fisher’s exact tests were employed for group comparisons. Survival analysis between two groups was visualized using the Kaplan–Meier plot, and the log-rank test was utilized to assess statistical significance. *p*-Value < 0.05 was considered significant. Statistical analyses were performed using R software (http://www.r-project.org/).

## Results

### Ki67 Gene Expression is Associated with Aggressive Cell Proliferation in ER+/HER2− BC

**Fig. 1:**
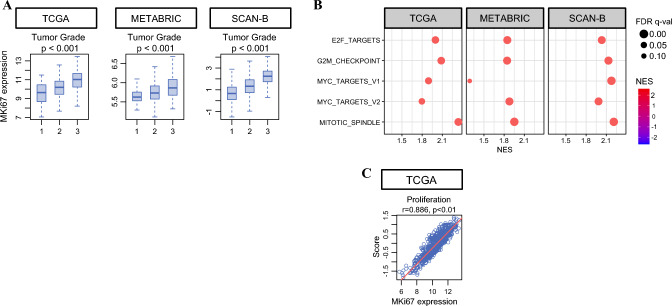
Association of *MKI67* expression with tumor grade and pathways related to cell proliferation, as well as correlation with proliferation score; **A** box plots showing *MKI67* expression across tumor grades in the TCGA, METABRIC, and SCAN-B cohorts (*p*-values from Wilcoxon rank-sum test); **B** dot plots from gene set enrichment analysis (GSEA) of the five gene sets that met the significance threshold of false discovery rate (FDR) < 0.25 in *MKI67*-high versus *MKI67*-low breast cancers in the TCGA, METABRIC, and SCAN-B cohorts; dot size represents the FDR *q*-value, and dot color indicates the normalized enrichment score (NES); **C** scatter plot illustrates the correlation between *MKI67* expression and proliferation score in the TCGA cohort (Pearson’s *r* = 0.886, *p* < 0.01)

Given that Ki67 assessed using IHC is one of the most commonly used markers of cell proliferation, we first validated whether *MKI67* expression correlates with other known cell proliferation markers in the cohorts we analyzed. As expected, *MKI67* expression significantly correlated with Nottingham histologic grade as a morphologic assessment of tumor aggressiveness, in patients with ER+/HER2− BC from three large independent cohorts (TCGA, METABRIC, and SCAN-B) (all *p* < 0.001, Fig. 1A). GSEA revealed that *MKI67*-high tumors significantly enriched all of the cell proliferation-related gene sets in the Hallmark collection, including E2F Targets, G2M Checkpoint, Mitotic Spindle, and MYC Targets v1 and v2 (all FDR < 0.25, Fig. 1B), consistent with the results for histologic grade. Importantly, the enrichment of proliferation-related hallmark gene sets remained significant when more stringent thresholds were applied (FDR < 0.05), confirming the robustness of our findings (Fig. S1A). In the TCGA cohort, the proliferation score was strongly correlated with *MKI67* expression (*r* = 0.886, Fig. 1C). We next examined the relationship between *MKI67* expression and intrinsic molecular subtype (PAM50) in cohorts with available annotations (TCGA, METABRIC, and SCAN-B). *MKI67* expression increased progressively across subtypes in the following order: normal-like, luminal A, luminal B, HER2-enriched, and basal-like (Fig. S1B). This pattern is consistent with established subtype biology and further supports transcriptomic Ki67 as a robust marker of tumor proliferative activity. These findings indicate that BCs with high *MKI67* expression exhibit enhanced proliferative activity.

### *MKI67*-High ER+/HER2− BC Trended to Have Worse Survival

**Fig. 2:**
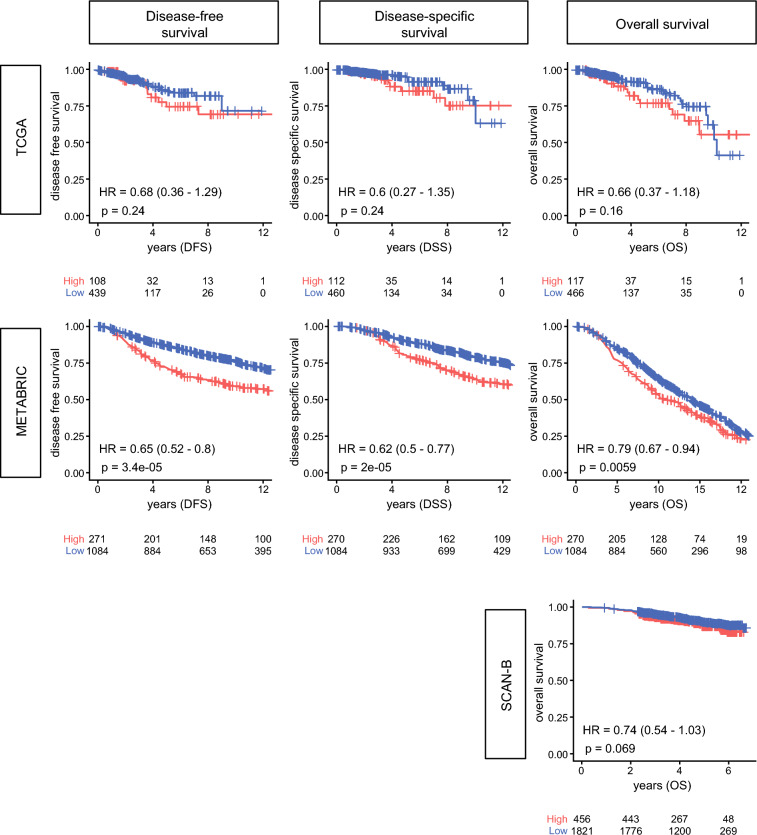
Association of *MKI67* expression with survival in TCGA, METABRIC, and SCAN-B cohort; Kaplan–Meier (KM) survival curves for disease-free survival (DFS), disease-specific survival (DSS), and overall survival (OS) in *MKI67*-high (red) versus *MKI67* low (blue) breast cancers from the TCGA and METABRIC cohorts, and KM survival curve for OS in SCAN-B cohort; hazard ratios (HRs) and *p*-values were calculated using the log-rank test

To assess whether this proliferative phenotype translated into patient outcomes, we next evaluated its prognostic impact on patient survival. High *MKI67* expression was associated with worse disease-free survival (DFS), disease-specific survival (DSS), and overall survival (OS) in the METABRIC cohort (Fig. 2). Similar trends for DFS, DSS, and OS were observed in TCGA and for OS in SCAN-B; however, these did not reach statistical significance.

### High *MKI67* Expression is Associated with Tumor Genomic Instability

**Fig. 3:**
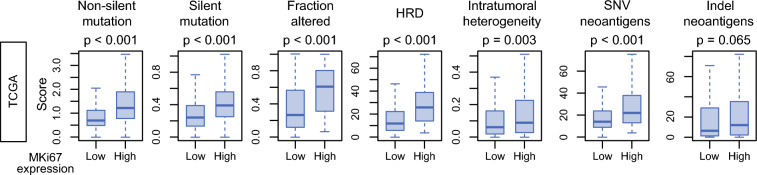
Association of *MKI67* expression with genomic instability and tumor immunogenicity-related features; box plots of *MKI67*-high versus *MKI67*-low breast cancers in the TCGA cohort, showing the estimated scores for non-silent mutation rate, silent mutation rate, fraction of genome altered, homologous recombination deficiency (HRD) score, intratumor heterogeneity, SNV-derived neoantigen count, and indel-derived neoantigen count; *p*-values were calculated using the Wilcoxon rank-sum test and error bars indicate the 95% confidence interval; center lines in boxes indicate the median, and box limits indicate the 25th and 75th percentiles

Given that high *MKI67* expression was strongly associated with increased proliferative activity, we next investigated its relationship with tumor genomic instability. In the TCGA cohort, *MKI67*-high tumors were significantly associated with higher genomic alteration metrics, including non-silent and silent mutation burden, fraction of the genome altered, and homologous recombination deficiency (HRD) (all *p* < 0.001; Fig. 3). *MKI67*-high tumors also exhibited increased intratumoral genomic heterogeneity (*p* = 0.003), SNV-derived neoantigens (*p* < 0.001), and indel-derived neoantigens (*p* = 0.065; Fig. 3). These findings suggest that highly proliferative ER+/HER2− BCs may harbor a more genetically unstable profile.

### *MKI67*-High Tumors Exhibit Greater Immune Cell Infiltration

**Fig. 4:**
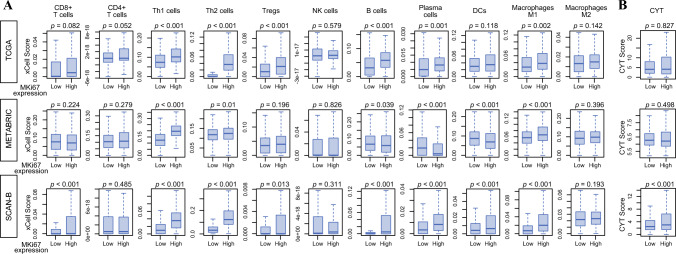
*MKI67* expression in relation to tumor immune cell infiltration and cytolytic activity (CYT) score in ER+/HER2− breast cancer. Box plots of *MKI67*-high versus *MKI67*-low breast cancers in the TCGA cohort, **A** showing immune cell populations (CD8+ T cells, CD4+ T cells, Th1 cells, Th2 cells, Regulatory T cells (Tregs), NK cells, B cells, Plasma cells, DCs, M1 macrophages, and M2 macrophages), and **B** CYT score. P-values were calculated using the Wilcoxon signed-rank test. Error bars indicate the 95% confidence interval; lines in boxes indicate the median, and box limits indicate the 25th and 75th percentiles

To further investigate the immune landscape associated with high *MKI67* expression, we examined its association with immune cell infiltration within the tumor microenvironment. We applied the xCell algorithm to bulk tumor transcriptomes from the TCGA, METABRIC, and SCAN-B cohorts to evaluate immune cell infiltration. Several immune cell types, including type 1 helper T cells (Th1), type 2 helper T cells (Th2), B cells, and type 1 (M1) macrophages, were significantly more abundant in *MKI67*-high tumors across all three cohorts (Fig. 4A; all *p* < 0.05), whereas other immune cell types did not show consistent or significant trends across the three cohorts. Cytolytic activity (CYT), calculated with expressions of perforin and granzyme B, did not associate significantly with *MKI67* expression in the TCGA and METABRIC cohorts, but was significantly higher in the *MKI67*-high tumors in the SCAN-B cohort (Fig. 4B; *p* < 0.001). While triple negative breast cancer (TNBC) tumors demonstrated overall higher immune infiltration, the association between *MKI67* expression and immune cell infiltration within TNBC was less pronounced and not as consistently observed as in ER+/HER2− disease (Fig. S4). These findings suggest that the relationship between proliferation and immune infiltration may vary by subtype rather than representing a uniform biological pattern across BC.

### *MKI67*-Low Tumors Enriched Multiple Gene Sets that Aggravate Cancer

**Fig. 5:**
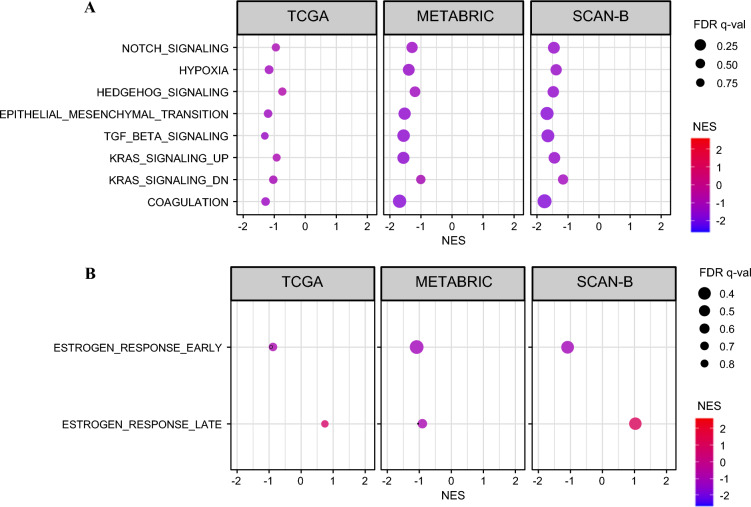
Hallmark pathway enrichment according to *MKI67* expression in ER+/HER2− breast cancer; **A** gene set enrichment analysis (GSEA) of selected hallmark pathways in *MKI67*-high versus *MKI*67-low tumors across TCGA, METABRIC, and SCAN-B cohorts; normalized enrichment score (NES) is shown on the *x*-axis; positive NES indicates enrichment in *MKI67*-high tumors, whereas negative NES indicates enrichment in *MKI67*-low tumors. Dot size represents FDR *q*-value, and color reflects NES magnitude and direction; **B** GSEA of hallmark ESTROGEN_RESPONSE_EARLY and ESTROGEN_RESPONSE_LATE gene sets across the same cohorts; most estrogen response pathway associations did not reach statistical significance (FDR *q*-values generally > 0.4) and should be interpreted as exploratory trends

Given that *MKI67*-high tumors were highly proliferative and genomically unstable but did not consistently translate into significantly worse survival, we next explored the biological features characterizing *MKI67*-low tumors. GSEA in the TCGA, METABRIC, and SCAN-B cohorts demonstrated that eight hallmark gene sets were significantly enriched in *MKI67*-low tumors (Fig. 5; all FDR < 0.25). This included hypoxia, epithelial–mesenchymal transition (EMT), and KRAS signaling pathways, all of which are associated with aggressive tumor biology. We additionally examined hallmark estrogen response gene sets. ESTROGEN_RESPONSE_EARLY showed negative normalized enrichment scores across all cohorts, indicating relative enrichment in *MKI67*-low tumors. ESTROGEN_RESPONSE_LATE demonstrated cohort-dependent patterns. However, most associations did not reach statistical significance (FDR > 0.4) and should be interpreted as trend-level findings. These results suggest heterogeneous estrogen signaling activity within *MKI67*-low tumors despite enrichment of hypoxia and EMT programs.

### Higher *MKI67* Expression was Associated with Pathological Complete Response (pCR) Following Neoadjuvant Chemotherapy (NAC)

**Fig. 6:**
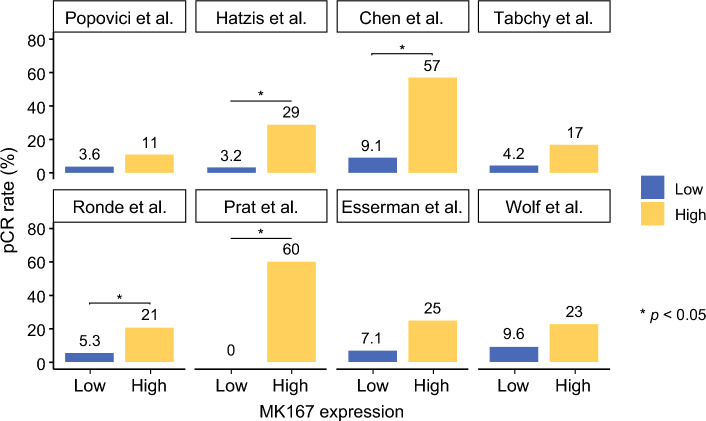
Association of *MKI67* expression with pCR rate after neoadjuvant chemotherapy (NAC); bar plots show the pCR rate in *MKI67*-high versus *MKI67*-low BCs across eight independent cohorts of patients treated with NAC; the included studies were: Popovici et al. (GSE20194, *n* = 140; paclitaxel, 5-fluorouracil, cyclophosphamide, and doxorubicin), Hatzis et al. (GSE25066, *n* = 274; doxorubicin and cyclophosphamide followed by taxane), Tabchy et al. (GSE20271, *n* = 89; 5-fluorouracil, doxorubicin, and cyclophosphamide or the same regimen with paclitaxel), Ronde et al. (GSE34138, *n* = 119; dose-dense doxorubicin and cyclophosphamide), Prat et al. (GSE50948, *n* = 25; doxorubicin/paclitaxel followed by cyclophosphamide/methotrexate/fluorouracil), Esserman et al. (GSE22226, *n* = 41; doxorubicin and cyclophosphamide with optional taxane), Chen et al. (GSE163882, *n* = 69; anthracycline and taxane with or without trastuzumab), and Wolf et al. (GSE180962, *n* = 65; standard anthracycline- and taxane-based chemotherapy); *p*-values were calculated using Fisher’s exact test, and * indicates *p* < 0.05

Next, we investigated the association between *MKI67* expression and response to chemotherapy using eight independent cohorts of patients with ER+/HER2− BC who received NAC (Fig. 6). Across the cohorts included in this analysis, patients received NAC consisting of paclitaxel, 5-fluorouracil, cyclophosphamide, and doxorubicin (TFAC) in Popovici et al. (GSE20194); doxorubicin/cyclophosphamide followed by taxane (ACT) in Hatzis et al. (GSE25066); anthracycline and taxane with or without trastuzumab in Chen et al. (GSE163882); 5-fluorouracil, doxorubicin, and cyclophosphamide or TFAC in Tabchy et al. (GSE20271); dose-dense doxorubicin and cyclophosphamide in Ronde et al. (GSE34138); epirubicin and cyclophosphamide followed by docetaxel in Prat et al. (GSE21974); doxorubicin and paclitaxel followed by cyclophosphamide, methotrexate, and 5-fluorouracil, with or without trastuzumab, in Esserman et al. (GSE50948); and standard anthracycline/taxane-based chemotherapy with or without ganitumab in Wolf et al. (GSE180962, I-SPY2). pCR rates were higher in *MKI67*-high tumors across all cohorts, with statistical significance observed in four of the eight cohorts (all *p* < 0.05). This finding validates the previous reports that highly proliferative tumors are more likely to respond to NAC and supports an association between *MKI67* expression and treatment response in ER+/HER2−BC.

## Discussion

High *MKI67* expression in ER+/HER2−BC was strongly associated with Nottingham histologic grade and enrichment of cell-cycle-related pathways. Although it trended toward worse survival outcomes, statistical significance was not consistently observed across cohorts. *MKI67*-high tumors demonstrated marked genomic instability, including higher mutation burden, a greater fraction of the genome altered, homologous recombination deficiency, increased neoantigen load, and greater intratumoral heterogeneity. They also exhibited enhanced immune infiltration by Th1, Th2, B cells, and M1 macrophages, though cytolytic activity varied across cohorts. Conversely, *MKI67*-low tumors were enriched for hypoxia, EMT, and KRAS signaling, suggesting alternative mechanisms of aggressiveness that may explain why their impact on survival outcomes was less pronounced than expected. Importantly, *MKI67*-high tumors achieved higher pCR rates following NAC in most cohorts, suggesting that high *MKI67* expression may serve as a predictive biomarker for NAC in ER+/HER2−BC.

Cell proliferation, as reflected by Ki67 expression, has long been linked to histologic grade and intrinsic molecular phenotype, and high-grade, highly proliferative tumors have consistently demonstrated greater sensitivity to NAC despite worse baseline prognosis.^7^^,^^39^ Consistent with this biology, Ki67 has been incorporated into multigene prognostic assays such as Oncotype DX and remains widely used in clinical practice.^7^ Immunohistochemical Ki67 expression correlates closely with tumor proliferation^39^ and is recognized as both a negative prognostic marker and a positive predictive biomarker for response to NAC.^6^^,^^7^ Ki67 is also commonly used to distinguish luminal A-like tumors from more proliferative luminal B-like tumors.^14^

Despite these established associations, we did not observe a marked difference in survival between *MKI67*-high and *MKI67*-low tumors in our analysis. Several factors may account for this observation. First, the transcriptomic Ki67 signal is not tumor-exclusive; bulk RNA profiling also captures *MKI67* transcripts from stromal and immune cells. Indeed, *MKI67*-high tumors demonstrated greater infiltration of Th1 and Th2 cells, B cells, and M1 macrophages, which may have partially mitigated the adverse prognostic impact of highly proliferative cancer cells. Second, treatment-mediated offset is likely, as *MKI67*-high tumors achieved higher pathologic complete response rates across multiple cohorts, indicating that chemosensitivity may attenuate or mask an otherwise negative baseline prognosis. Taken together, these opposing biological and treatment-related factors likely contribute to the absence of clear survival separation when tumors are stratified solely by *MKI67* expression.

High Ki67 expression has been associated with improved response to chemotherapy,^9^^,^^10^ yet it also characterizes biologically aggressive tumors that are more likely to recur and lead to worse outcomes.^6^^,^^40^ This can be partially explained by the underlying genomic features of *MKI67*-high tumors observed in our results. These tumors demonstrated greater genomic instability, with elevated HRD scores, higher mutation burden, and increased neoantigen load, all hallmarks of an aggressive phenotype. Such characteristics, including significantly high intratumoral genomic heterogeneity, may promote the survival and expansion of resistant clones, ultimately leading to recurrence and worse survival outcomes. Furthermore, despite increased chemosensitivity, tumors with high *MKI67* expression exhibit worse long-term survival outcomes. This pattern is consistent with prior studies^6^^,^^9^^,^^10^ that have identified a disconnect between initial response and sustained disease control.

In addition to genomic instability, the tumor immune microenvironment was activated in highly proliferative tumors. *MKI67*-high tumors exhibited a more immunogenic phenotype, characterized by elevated infiltration of tumor-infiltrating lymphocytes and immune cell subsets including Th1/Th2 cells, M1 macrophages, regulatory T cells, B cells, and plasma cells. These results are consistent with previous reports,^7^^,^^40^ and this increased immune infiltration is expected to contribute to antitumor activity.

By contrast, *MKI67*-low tumors were enriched for pathways associated with hypoxia, epithelial–mesenchymal transition (EMT), KRAS signaling, and coagulation. These molecular programs are linked to therapy resistance and a mesenchymal or stem-like phenotype. This finding may also partially explain why tumors with high *MKI67* expression do not consistently exhibit worse survival outcomes across all cohorts, as both high and low *MKI67* expressing tumors are associated with tumor-aggravating factors. This observation also aligns with prior publications suggesting that low-proliferation ER+/HER2− tumors may benefit from biologically guided, non-cytotoxic regimens.^17^^,^^41^ These data suggest that transcriptomic *MKI67* expression provides additional biological insight beyond conventional Ki67 immunohistochemistry. It captures not only proliferative activity, but also genomic and immunologic features that may influence both short- and long-term outcomes. Given the well-documented variability of Ki67 measurement using IHC, including inconsistencies in antibody selection, scoring methods, and sample handling,^15^^,^^16^ transcriptomic quantification of Ki67 expression may offer a more reproducible and standardized approach for clinical application.

Taken together, these findings highlight *MKI67* expression as a quantitative biomarker that integrates biological features with predictive significance in ER+/HER2−BC. Its strong association with proliferative activity, genomic instability, and immune infiltration provides additional biological insight beyond conventional IHC-based assessment. Prospective validation studies are warranted to establish clinically applicable thresholds and confirm its role in treatment decision-making.

This study has several limitations. First, it is retrospective in nature, and our findings require validation in prospective clinical trials. This is also a correlative study and is not intended to demonstrate mechanisms. Functional studies are needed to directly validate the mechanisms inferred from transcriptomic associations, including the relationship between immune activation, genomic instability, and outcomes. Second, although we integrated multiple datasets and platforms, differences in cohort composition, treatment regimens, and follow-up duration may introduce bias. Third, while *MKI67* expression was used as a surrogate for tumor proliferation, it is only one component of a complex biological landscape, and additional markers may be necessary to fully characterize treatment responsiveness and prognosis. Fourth, the use of a within-cohort, rank-based *MKI67* classification reflects underlying differences in expression scaling across platforms and precludes definition of a universal, individual-level cutoff, highlighting the need for standardized normalization and prospective validation before clinical implementation. Fifth, immune profiling variables were not uniformly available across all neoadjuvant cohorts, limiting our ability to perform consistent multivariable modeling to disentangle the relative contributions of tumor-intrinsic proliferation and immune infiltration to pCR. As a result, the extent to which the observed *MKI67*–pCR association reflects direct proliferative biology versus immune-mediated effects could not be fully quantified in this study. Sixth, while *MKI67* expression was used as a surrogate for tumor proliferation, matched IHC-based Ki67 data were not available across cohorts, precluding direct comparison of predictive performance or formal optimization of clinically actionable thresholds. Accordingly, additional prospective studies incorporating head-to-head comparisons with established proliferation markers and robust performance metrics will be required to define the clinical utility of transcriptomic *MKI67*. Finally, intrinsic molecular subtype PAM50 data were not uniformly available across all included cohorts, precluding systematic subtype-stratified analyses for every dataset. Although subtype distribution was examined in TCGA, METABRIC, and SCAN-B, we were unable to comprehensively adjust for intrinsic subtype across all cohorts. Given the known discordance between receptor-defined subtype and intrinsic molecular subtype, partial enrichment of basal-like tumors within the highest *MKI67* stratum cannot be completely excluded. Because basal-like tumors exhibit higher immune infiltration and greater responsiveness to NAC, this may have contributed to the observed associations. Future studies incorporating standardized intrinsic subtype annotation across NAC cohorts will be important to further disentangle proliferation-driven effects from subtype-specific biology.

## Conclusions

Our study highlights the biological and associative relevance of *MKI67* at the transcriptomic level in relation to neoadjuvant treatment response in ER+/HER2−BC. By validating these associations across multiple large, independent cohorts, we demonstrate the robustness and reproducibility of transcriptomic *MKI67* assessment.

## Supplementary Information

Below is the link to the electronic supplementary material.

Supplementary file1 (DOCX 379 KB)
