# Supplementary material for: Ki67 Gene Expression is Associated with Immune Cell Infiltration and Neoadjuvant Chemotherapy Response in ER+/HER2− Breast Cancer
Source: Ann Surg Oncol. 2026 Apr 10;33(7):6422–32. doi: 10.1245/s10434-026-19620-2 (PMC13242411; doi:10.1245/s10434-026-19620-2)
Supplement: Supplementary file 1 — Supplementary file1 (DOCX 379 KB) [file 10434_2026_19620_MOESM1_ESM.docx]

| **Cohort Name** | **Author** | **Year** | **Sample Size** | **Treatment Regimen** | **Platform** |
| --- | --- | --- | --- | --- | --- |
| GSE20194 | Popovici | 2010 | 140 | Paclitaxel + 5-fluorouracil, AC | Affymetrix U133A microarray |
| GSE25066 | Hatzis | 2011 | 274 | Taxane and anthracycline-based | Affymetrix U133A microarray |
| GSE163882 | Chen | 2022 | 69 | Taxane and anthracycline-based | Illumina NextSeq 500 |
| GSE20271 | Tabchy | 2010 | 89 | Paclitaxel, fluorouracil, AC | Affymetrix U133A Array |
| GSE34138 | Ronde | 2013 | 119 | Dose-dense AC  → docetaxel/capecitabine if poor MRI response | Illumina HumanWG-6 v3 |
| GSE50948 | Prat | 2014 | 25 | Doxorubicin/paclitaxel followed by cyclophosphamide/methotrexate/fluorouracil | Affymetrix U133A Array |
| GSE22226 | Esserman | 2012 | 41 | AC with optional taxane | Agilent Whole Human Genome  4×44K |
| GSE180962 | Wolf | 2021 | 65 | Paclitaxel → AC | Agilent DiscoverPrint (15746)  Agendia 32K DP v1.14 (SCFG+) |
| **Abbreviation: AC**: Doxorubicin (Adriamycin) + Cyclophosphamide, **MRI**: Magnetic resonance imaging | | | | | |

Supplementary Table 1. Characteristics of neoadjuvant chemotherapy cohorts included in the analysis.

**
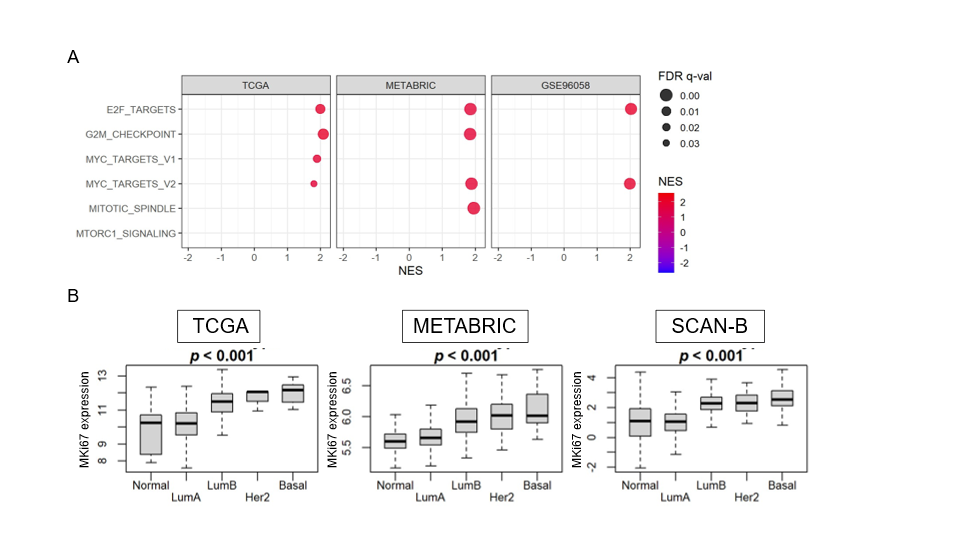
**

**Supplementary Figure S1: *MKI67* expression in relation to hallmark pathway enrichment and PAM50 intrinsic subtypes. (A)** GSEA comparing *MKI67*-high and *MKI67*-low tumors (*FDR* < 0.05). **(B)** Association of *MKI67*expressions with PAM50 intrinsic breast cancer subtypes. Box plots showing *MKI67* expression across tumor grades in the TCGA, METABRIC, and SCAN-B cohorts (*p* values from Wilcoxon rank-sum test).

**
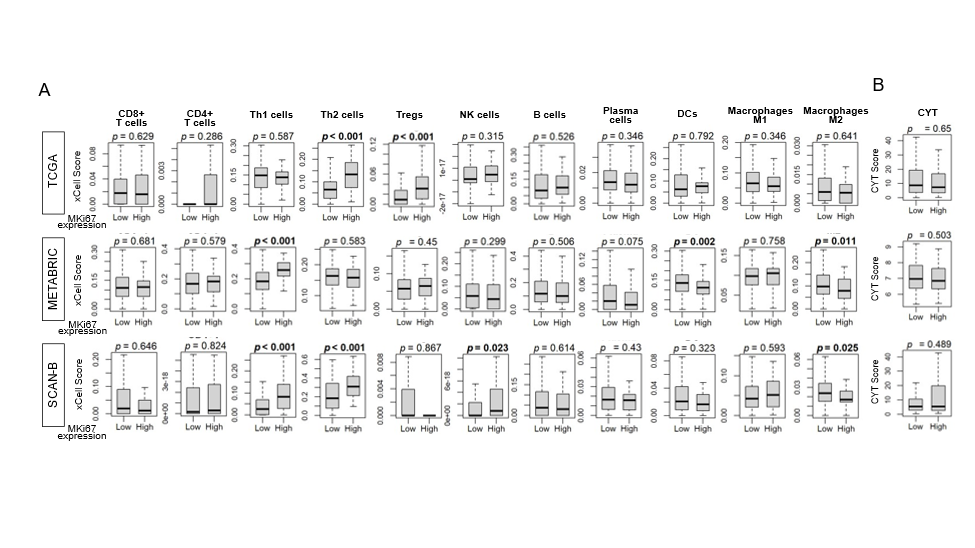
**

**Supplementary Figure S4:** *MKI67* expression in relation to tumor immune cell infiltration and cytolytic activity (CYT) score in triple negative breast cancer. Box plots of MKI67-high versus MKI67-low breast cancers in the TCGA cohort, (A) showing immune cell populations (CD8+ T cells, CD4+ T cells, Th1 cells, Th2 cells, Regulatory T cells (Tregs), NK cells, B cells, Plasma cells, DCs, M1 macrophages, and M2 macrophages), and (B) CYT score. P-values were calculated using the Wilcoxon signed-rank test. Error bars indicate the 95% confidence interval; lines in boxes indicate the median, and box limits indicate the 25th and 75th percentiles.
